# Treatment of Phosphorus Poisoning

**Published:** 1892-02

**Authors:** 


### TREATMENT OF PHOSPHORUS POISONING.

Dr. Bokai (*Le Bulletin Medical*, No. 100, 1891,) communicated a new method of treating phosphorus poisoning. The use of turpentine and the salts of copper, give a mortality of 50 to 60 per cent. The writer has found a solution of potassium permanganate, two to five grammes (thirty grains to one and a half drachms) in one thousand parts of water, to form a chemical antidote. The oxygen of this compound is liberated and unites with the phosphorus to form ortho-phosphoric acid, which is inoffensive. The same reaction takes place in the stomach, and, what is more, the oxide of manganese is transformed into a chloride, and the quantity of oxygen liberated is abundantly sufficient to oxydize the phosphorus present.

Experiments on dogs have demonstrated the efficacy of this treatment; those treated thus, after poisoning with large doses recovered, while the animals used to control the experiment all perished.—*The Cincinnati Lancet-Clinic.*

